# Supplementary material for: Influence of plastic film mulch with biochar application on crop yield, evapotranspiration, and water use efficiency in northern China: A meta-analysis
Source: PeerJ. 2021 Mar 3;9:e10967. doi: 10.7717/peerj.10967 (PMC7936560; doi:10.7717/peerj.10967)
Supplement: Supplemental Information 3 [file peerj-09-10967-s003.docx]

Meta-analysis rationale

1. The rationale for conducting the meta-analysis

Extended seasons of aridity and water deficiency caused by reduced frequency and magnitude of rainfall events worsened by changes in climatic conditions affect soil water availability across the world. Mulching has been used as essential agronomic practice for sustaining rain-fed agriculture, predominantly in semi-arid areas. Mulching is also an eco-friendly soil water management practice that retards evapotranspiration, and improves water use efficiency and yield of crops. That notwithstanding, plastic film as a mulch material has been highly patronized in China. However, varied reports of the influence of co-application of plastic mulch and biochar on crop yield, water use efficiency and evapotranspiration is gaining prominence in agronomic research in recent times. Whiles others report of the co-application of plastic film with biochar as useful for improving soil and water conservation in rain-fed agriculture, recent studies have suggested co-application of plastic mulch and biochar as an integrated practice to avert drought while simultaneously mitigating runoff and erosion.
