# Supplementary material for: Influence of plastic film mulch with biochar application on crop yield, evapotranspiration, and water use efficiency in northern China: A meta-analysis
Source: PeerJ. 2021 Mar 3;9:e10967. doi: 10.7717/peerj.10967 (PMC7936560; doi:10.7717/peerj.10967)
Supplement: Supplemental Information 4 [file peerj-09-10967-s004.docx]

2. The contribution that it makes to knowledge in light of previously published related reports, including other meta-analyses

The effects of plastic film mulch and biochar on crop yield, ET, and WUE in rain-fed agriculture in China is a growing area of research. We therefore comprehensively reviewed relevant and current literature on how co-application of plastic film mulching with biochar influence crop yield, WUE and ET. This serves as useful resource for study on impact of co-application of plastic mulch and biochar on crop yield, WUE and ET particularly in rain-fed agriculture in northern China. The PRISMA guideline for meta-analysis was followed in selecting articles for the analysis (Moher et al., 2009).
